# Advancements in AI-based quantitative analysis of fundus tessellation and its application in myopia research

**DOI:** 10.3389/fmed.2026.1786949

**Published:** 2026-03-13

**Authors:** Yi-Ming Guo, Tian Zhan, Jing Zheng, Junhan Wei, Jiaqi Wang, Yijin Han, Juan Huang, Xingye Wang, Guoyun Zhang, Lu Ye

**Affiliations:** 1 Shaanxi Eye Hospital, Xi’an People’s Hospital (Xi’an Fourth Hospital), Affiliated People’s Hospital of Northwest University, Xi’an, China; 2 EVision Technology (Beijing) Co. Ltd., Beijing, China

**Keywords:** artificial intelligence, axial length, fundus tessellation, fundus tessellation density, myopia

## Abstract

**Background:**

Fundus tessellation (FT) is increasingly recognized as an early structural manifestation of retinal and choroidal remodeling in myopia, reflecting initial changes associated with axial elongation. With advances in artificial intelligence (AI), particularly deep learning–based image analysis, quantitative assessment of FT has emerged as a promising approach for objective and scalable evaluation in myopia research.

**Methods:**

This review provides an integrative overview of recent studies applying AI-assisted and quantitative image-analysis approaches to fundus tessellation assessment. Relevant literature was identified through a structured search of major biomedical databases, focusing on methodologies for FT and fundus tessellation density (FTD) quantification and their reported associations with clinical ocular parameters in myopia.

**Results:**

Across multiple cohorts, AI-derived FTD consistently showed associations with axial length elongation, choroidal thinning, and increasing myopia severity. Common analytical frameworks involved region-of-interest definition, image normalization, and supervised deep learning–based segmentation. Several studies further reported spatial heterogeneity in FTD distribution and its relationship with peripapillary and macular alterations, supporting the potential role of FT-related metrics as quantitative imaging biomarkers for myopic structural change.

**Conclusion:**

AI-driven quantification of fundus tessellation represents a methodological advancement in myopia research by enhancing objectivity and scalability in retinal image analysis. These approaches may facilitate early risk stratification and hold promise for future longitudinal assessment of myopia.

## Introduction

Myopia, a common refractive disorder of increasing global concern, is characterized by earlier onset and greater severity in recent decades. By 2050, nearly 50% of the global population is projected to be affected by myopia, including over one billion cases of high myopia (1). Among these, pathological myopia accounts for approximately 50%–70% (2, 3), representing a major contributor to visual impairment and posing a considerable socioeconomic burden (4). Progressive pathological changes in myopia compromise visual function and significantly increase the risk of severe posterior segment complications, including posterior scleral staphyloma (5), retinal detachment (6), macular hole formation (7), and choroidal neovascularization (8). In advanced stages, such complications may culminate in irreversible vision loss (9). Progressive structural degeneration—particularly involving retinal and choroidal tissues—plays a pivotal role in the pathophysiology of high myopia, serving as a foundation for monitoring disease trajectory and guiding clinical intervention (10). Consequently, these microstructural alterations have garnered significant attention in current ophthalmic research.

Accurate evaluation of retinal architecture is paramount for diagnosing and managing ocular conditions. However, conventional diagnostic methods, which predominantly rely on manual interpretation, suffer from significant limitations in terms of efficiency, objectivity, and reproducibility—particularly when applied to the recognition and quantification of early and subtle structural abnormalities (11, 12). The rapid evolution of deep learning, image segmentation, and computer vision has led to the growing application of artificial intelligence (AI) in medicine, advancing from rudimentary screening tools to sophisticated systems capable of autonomous recognition and clinical decision support (13, 14). In ophthalmology—a field inherently dependent on high-resolution imaging—AI-driven algorithms offer new paradigms for enhancing traditional diagnostic workflows. Although AI-based deep learning models have demonstrated promising accuracy in detecting various retinal diseases (15), their application in myopia, especially in evaluating retinal alterations associated with pathological myopia, remains in its nascent stage. However, this limited application contrasts sharply with the substantial epidemiological burden of myopia in East Asia, where prevalence among South Korean students has reached 73%, with high myopia at 5% (16), and rates in Japan rise to 94.9% among high school students, with high myopia at 11.3% (17). These patterns highlight an urgent need for scalable, objective, and automated quantitative tools capable of supporting early detection and longitudinal monitoring in large myopic populations. As such, integrating intelligent systems into the precise assessment of myopia-related structural changes has emerged as a critical research priority.

Fundus tessellation (FT) is increasingly recognized as one of the earliest observable structural alterations in myopic eyes because it directly reflects the initial remodeling of the choroid–sclera complex during axial elongation (18). As the globe elongates, the posterior sclera undergoes biomechanical stretching and extracellular matrix remodeling, which secondarily leads to progressive choroidal thinning and attenuation of the retinal pigment epithelium. These changes increase the visibility of large choroidal vessels through the increasingly transparent overlying tissues, giving rise to the characteristic mosaic-like appearance of FT. Importantly, such alterations precede the development of more advanced lesions—including diffuse atrophy, lacquer cracks, and posterior staphyloma—therefore positioning FT as a sensitive early structural biomarker that captures the onset of posterior pole stress and choroidal perfusion decline. (19, 20).

However, in individuals with myopia, excessive axial elongation leads to retinal distortion, choroidal thinning, and irregularities in the retinal pigment epithelium, significantly reducing the accuracy of conventional image segmentation algorithms (21). While preliminary FT grading systems have been introduced (22), their clinical applicability remains limited due to poor adaptability to phenotypic heterogeneity and weak correlations with key risk factors, which hinders their effectiveness in personalized clinical evaluations (23). Therefore, the development of FT quantification models that provide higher accuracy and consistency has become a critical focus in current myopic fundus imaging research.

In light of these challenges, the development of accurate and reproducible FT quantification models is a key focus in myopia-related retinal imaging research. This review highlights recent advancements in AI-based FT analysis, discusses current limitations, and suggests future directions for further research and clinical application.

## Methods

A literature search was conducted in PubMed, Embase, and Web of Science to identify studies addressing artificial intelligence–based quantitative assessment of fundus tessellation and FTD in myopia research. Publications available from database inception through October 2025 were considered. Search terms encompassed “fundus tessellation,” “fundus tessellation density,” and “myopia,” combined with terminology related to artificial intelligence methodologies, including “deep learning,” “machine learning,” “convolutional neural network,” “computer vision,” and “image segmentation.”

The initial search yielded 142 records. After removal of duplicates and screening of titles and abstracts for relevance, 25 articles were deemed eligible for full-text review. Eligible studies were those that reported quantitative AI- or machine learning–based approaches for FT or FTD analysis and examined their associations with myopia-related clinical parameters. Studies limited to purely technical algorithm development without ophthalmic application, conference abstracts lacking complete methodological description, animal studies, and publications without direct relevance to myopia were excluded. Within the eligible literature, studies considered representative were selected on the basis of methodological rigor, innovation, clinical relevance, and their contribution to advancing quantitative FT assessment in myopia research.

Given the diversity of study designs and analytical frameworks across the included publications, the present review provides a narrative and conceptual synthesis rather than a formal systematic evaluation.

### The relationship between tessellated fundus and myopia progression

Myopia progression is frequently accompanied by structural alterations in the retina, including macular degeneration and optic nerve damage, which contribute to its global status as a leading cause of irreversible visual impairment. To enable standardized assessment of myopia-related retinal pathology, the International Photographic Classification and Grading System for Myopic Maculopathy (META-PM) has established a widely accepted grading framework (24). Within this framework, fundus tessellation is designated as Category 1 (C1), representing the earliest retinal alteration. It is characterized by a mosaic-like appearance resulting from the enhanced visibility of large choroidal vessels through the thinned retinal and choroidal layers in the posterior pole.

Notably, although FT often coexists with other structural alterations in myopic eyes, it represents a distinct and much earlier manifestation of posterior pole remodeling. For instance, peripapillary atrophy (PPA) reflects atrophic changes surrounding the optic disc and is strongly associated with optic disk tilt and parapapillary structural stretching rather than early choroidal exposure (25). Choroidal thinning, typically assessed using OCT, denotes a quantitative reduction in choroidal thickness with axial elongation; however, thinning alone does not generate the tessellated appearance unless the overlying RPE becomes sufficiently attenuated to reveal underlying choroidal vessels (26). In comparison, diffuse chorioretinal atrophy (DCA) corresponds to a more advanced stage of myopic maculopathy (META-PM Category 2), characterized by broad RPE and choroidal loss and therefore failing to capture the subtle early-stage remodeling seen in younger or less advanced myopic eyes (27). Against this background, FT serves as a transition point between normal retinal appearance and overt pathological changes. Rather than indicating structural loss, FT reflects increased visibility of choroidal vasculature due to early RPE transparency alteration and posterior scleral expansion. This places FT uniquely at the initial phase of the myopic degeneration continuum, allowing detection of microstructural remodeling before the onset of irreversible atrophic lesions.

Fundus tessellation is commonly observed in individuals with myopia, with its prevalence varying according to region and refractive status, particularly in East Asia, where myopia is highly prevalent and the occurrence of FT has been extensively investigated. In Japan, Yoshihara examined 100 healthy adults aged 22–39 years and found that 43 individuals exhibited tessellated fundus, with 16 classified as strongly tessellated (28). Subsequently, Terasaki reported a 65.1% prevalence of FT in a cohort of 126 healthy volunteers (mean age 26.0 years; age range 22–39), with the temporal-inferior quadrant most commonly affected (29). Yamashita conducted an analysis of 1,670 right eyes from participants aged 40 and older in Kumejima, observing no FT in 911 eyes. Among the remaining, FT was most prevalent in the peripapillary region (383 eyes), followed by the inferior region (239 eyes) (30). In mainland China, Guo et al. reported a 48.1% prevalence of FT (95% CI: 45.7%–50.7%) among 1,443 adolescents aged 9–16 years (24). A 2021 study conducted in Shanghai involving 513 children and adolescents with high myopia (mean age 13.47 years; mean spherical equivalent: −8.34 D) revealed that only 5.7% of eyes exhibited no FT changes (29). In the same year, another investigation conducted on university students found that 90.2% of eyes (718/796) exhibited FT, including 447 cases of mild myopia and 196 of high myopia (31). Recently, Huang reported a FT prevalence of 42.18% in a cohort of 1,062 children in Nanjing (mean age 7.38 years; spherical equivalent ranging from −3.88 to −4.50 D) (32). Collectively, these findings underscore the high prevalence of FT across different age groups and degrees of myopia.

Beyond prevalence, numerous studies have investigated the relationship between FT and various ocular parameters. Terasaki et al. (25) reported that eyes with FT had significantly longer axial lengths compared to those without. Further analyses revealed that the retinal distribution of FT was associated with axial elongation, with FT in the posterior pole associated with greater axial length than FT in the parapapillary or inferior regions (30). Yoshihara et al. quantified FT severity using tessellated fundus indices (TFIs) and found significant correlations between TFIs and both subfoveal and nasal choroidal thickness, suggesting that choroidal thinning may contributes to the development of FT (28). Large-scale cohort studies have also identified associations between FT and a range of demographic and clinical variables, including age, sex, body mass index (BMI), best-corrected visual acuity, presence of parapapillary beta zone, and the prevalence of intermediate or late-stage age-related macular degeneration (AMD) (22). In pediatric myopic populations, FT severity exhibits a strong correlation with the degree of myopia. Gong et al. conducted a screening study involving 1,127 children with low myopia and found that 591 cases (52.4%) exhibited FT. The severity of FT was associated with a gradual reduction in choroidal thickness and was independently correlated with a larger corneal curvature radius (CR) (22). Furthermore, FT in myopic individuals may progress to advanced stages of myopic maculopathy, including lacquer cracks and diffuse atrophy (33). In a longitudinal cohort study conducted in Beijing from 2001 to 2011 involving 2,695 individuals, 19% of highly myopic eyes with baseline FT developed myopic maculopathy during follow-up (34). Similarly, in a 12.7-year longitudinal study, Hayashi et al. observed that among 276 eyes with FT, 28 (10.1%) progressed to diffuse chorioretinal atrophy, 8 (2.9%) developed lacquer cracks, and 1 (0.4%) progressed to choroidal neovascularization (35).

In summary, FT is highly prevalent in myopic individuals and strongly associated with refractive error severity and ocular structural parameters. As an early indicator of myopia progression, FT evaluation offers valuable insight in assessing and managing myopic retinal changes.

### AI and quantification of fundus tessellation

The increasing demand for ophthalmologists, combined with variability in the consistency and sensitivity of retinal image interpretation, has led to a surge in the application of artificial intelligence technologies in ophthalmology. AI has proven to be a critical tool in enhancing diagnostic accuracy and efficiency, particularly in the detection of various ophthalmic diseases such as glaucoma (36), age-related macular degeneration (37, 38), and diabetic retinopathy (39, 40). Tan et al. (41)introduced a ten-layer convolutional neural network (CNN) framework for automatic segmentation of exudates, microaneurysms, and hemorrhages in fundus images, demonstrating high sensitivity. Following this, Lam et al. (42) used the EyePACS database to develop a CNN that accurately detected red lesions (microaneurysms and exudates), achieving an area under the curve (AUC) of 0.94–0.95, further confirming the robustness of deep learning in detecting retinal lesions (43). Building on these findings, AI applications have progressively extended beyond traditional retinal diseases to a broader spectrum of ocular conditions, particularly demonstrating significant potential in the early detection of myopia and its associated pathological changes. While AI applications have been explored for identifying and risk-stratifying myopic retinal alterations, such as choroidal atrophy, Fuchs spots, and posterior staphyloma (44), and predicting myopia progression from fundus images (45), existing studies have primarily focused on classification tasks. There remains a notable gap in the quantitative analysis of myopia-related retinal changes, particularly for objective measures such as FT. For instance, Pan et al. developed a deep learning system based on Inception V3 and ResNet-50, which successfully classified normal fundus images, age-related macular degeneration, and FT. However, this study did not focus on myopic populations, and its classification merely confirmed the presence of FT without offering detailed quantification of FT severity (46). Conversely, Jiang et al. graded FT in myopic patients using the ETDRS grid, exploring its correlation with contrast sensitivity and preoperative refractive status. While this study achieved more detailed grading through subjective scoring, it still relied on manual interpretation and did not employ automated, standardized quantitative analysis (47). Given the critical importance of FT assessment in monitoring myopia progression, this section reviews recent advancements in quantitative analysis methods for FT in myopic patients, highlighting the potential and challenges of integrating FT quantification into fundus image analysis.

### Quantitative analysis of RGB image information in myopic fundus changes

The chromatic characteristics of fundus images are primarily determined by the selective absorption of visible light by different retinal and choroidal tissues. As incident light traverses the ocular media and reflects from posterior segment structures, differences in light absorption by various pigments within these tissues alter the spectral composition of the reflected light, thereby influencing the final color representation in the image (48, 49). The retinal pigment epithelium (RPE) and choroid contain high concentrations of melanin, while the vascular system primarily contains hemoglobin. Melanin, although having broad absorption capabilities, minimally influences the color presentation in fundus images due to its uniform absorption across wavelengths. Hemoglobin, however, exhibits strong wavelength-dependent absorption, especially in the blue (450–495 nm) and green (495–570 nm) ranges, significantly impacting the color composition of reflected light. In regions with high hemoglobin content, the reflected light predominantly appears red, followed by green, with blue being the least. In contrast, areas with lower hemoglobin content are characterized by a predominance of green and blue in the reflected light (50). Consequently, the variation in light absorption and reflection properties directly influences the color composition in fundus images, making the spectral absorption characteristics of hemoglobin a key factor in determining the retinal reflection tone. As such, conventional fundus imaging relies on the relative abundance of RGB pixels in the image to represent the variations in color due to these absorption properties. Thus, the relative number of RGB pixels in a fundus image quantifies its color composition. For instance, (51) analyzed fundus images of 39 patients with Vogt–Koyanagi–Harada (VKH) disease, using the ratio R/(R + G + B) to quantify the degree of the “sunset glow” fundus, a reflection of pigment loss. This index was significantly correlated with disease duration and melanocyte reduction (51). Similarly, in 2013, the Laguna ON_*h*_E program was employed to analyze color information from the optic nerve head (ONH) region using color fundus images to assess local hemoglobin content. The program, based on image analysis algorithms, first performs semi-automated segmentation of the ONH boundary and identifies the central retinal vascular area. It then extracts the reflectance intensity of the red, green, and blue spectral components from the image and constructs several color formulas [such as R−G, R−B, R−(G + B), (R−G)/R, (R−G)/G, etc.,] to approximate the hemoglobin concentration in a nearly linear manner (52). This method enables semi-quantitative analysis of tissue hemoglobin content by normalizing the color indices of each pixel in the ONH region against those of the corresponding vascular areas (50).

In myopia, Neelam adapted Suzuki’s method to construct the myopic chorioretinal degeneration index (MCDI) based on 152 color fundus images, revealing a significant positive correlation with axial length and visual acuity (53). In 2014, Yoshihara et al. developed a tessellated fundus index (TFI) to quantitatively assess tessellated changes in the fundus of non-pathological myopia patients. Using ImageJ, they extracted RGB pixel information from a specific region between the fovea and the optic disk to construct RGB color histograms. Three TFI values were calculated: TFI−1 = (R−G)/R, TFI−2 = R/(R + G + B), and TFI−3 = (R−G)/(R + G + B). The results showed that all TFI values exhibited excellent reproducibility both between evaluators and in repeated measurements, and were significantly correlated with the subjective grading of tessellated changes observed in fundus images (28).

In conclusion, RGB-based quantitative indices enhance the objective assessment of pigment and hemoglobin distribution in the retina and choroid, providing reproducible and reliable parameters for analyzing myopia-related tessellated changes. These indices hold considerable promise for both future research and clinical applications in the assessment of myopic fundus changes.

### Application of artificial intelligence and image processing in myopic fundus texture analysis

Advances in image processing and computer vision have facilitated region-of-interest (ROI)-based analytical techniques capable of detecting subtle textural variations in fundus images that are frequently imperceptible through conventional clinical evaluation (54). The integration of deep learning methodologies, particularly Convolutional Neural Networks (CNNs), has facilitated the development of automated image processing systems capable of extracting complex features from large-scale image datasets without reliance on manual descriptors (55, 56). These innovations have laid a methodological foundation for objectively quantifying the spatial distribution of FT in retinal images, thereby advancing the phenotypic analysis of myopia-associated fundus alterations.

**FIGURE 1:**
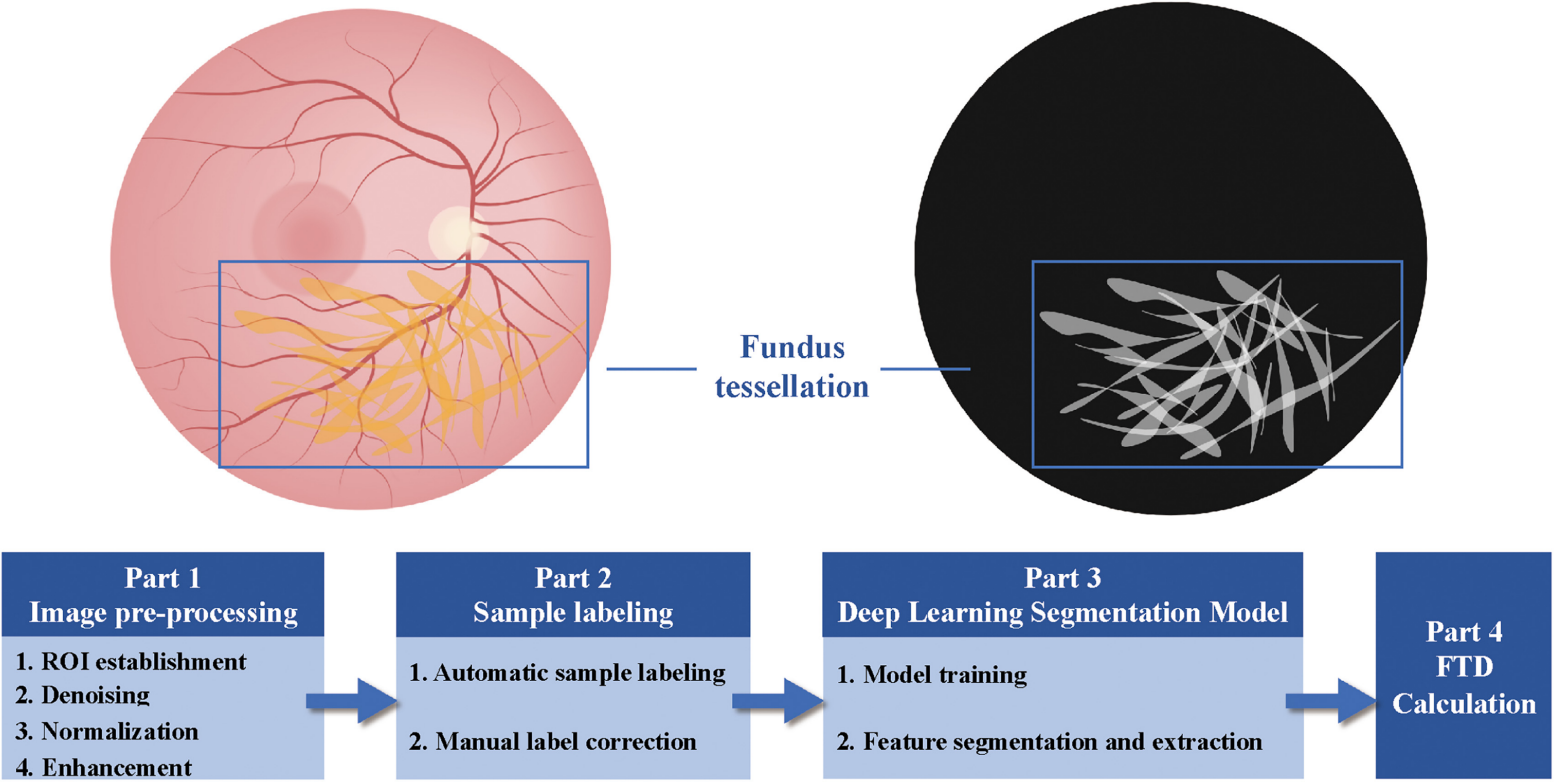
Schematic representation of the AI-based workflow for quantitative analysis of fundus tessellation.

**FIGURE 2:**
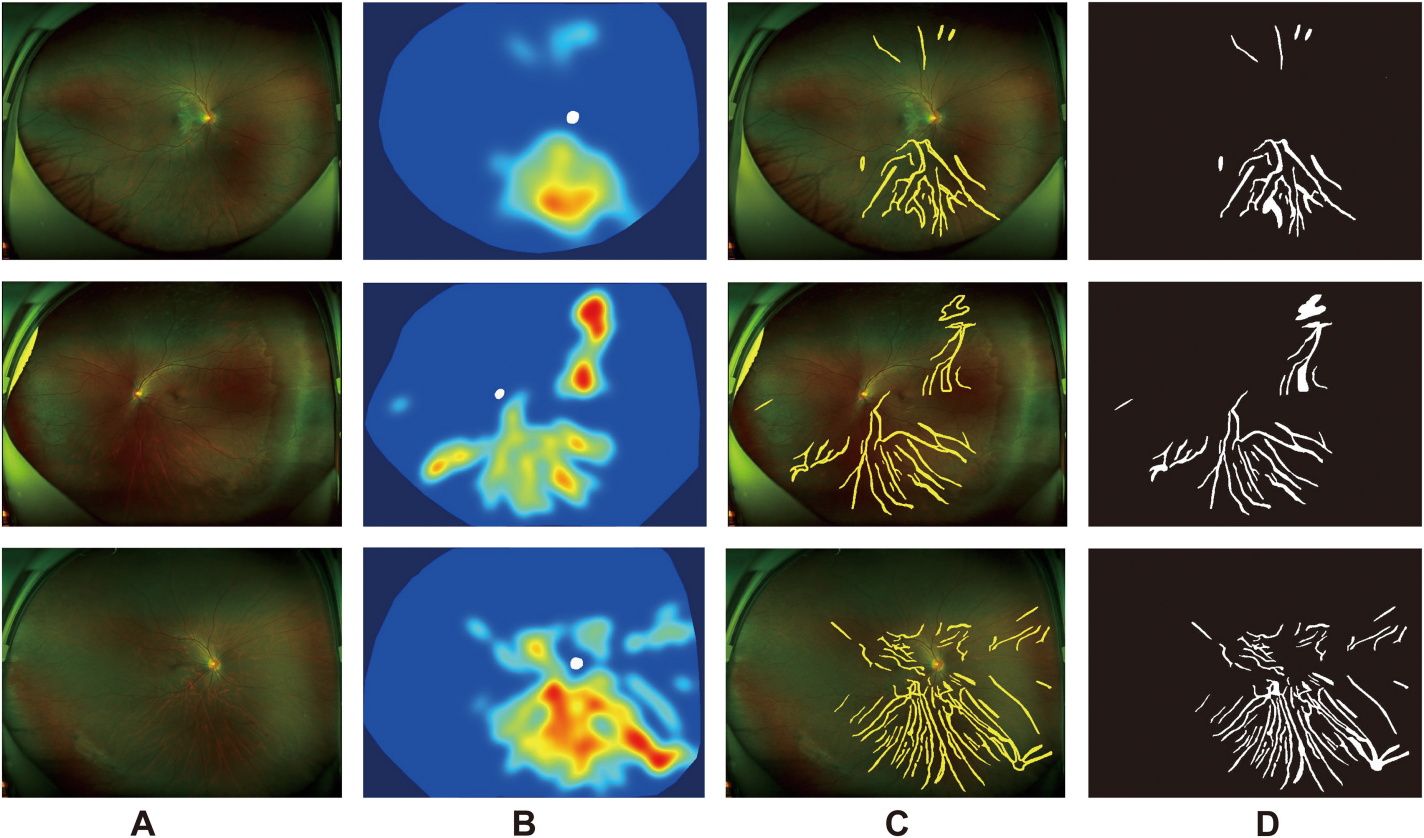
Quantitative visualization of fundus tessellation density across different severities of myopia. Rows show representative fundus photographs from eyes with mild, moderate, and high myopia, respectively. Columns **(A–D)** illustrate the sequential processing steps for FTD analysis, including the original color fundus image **(A)**, the model-generated FTD heatmap in which the color scale represents the relative intensity of tessellated regions corresponding to choroidal visibility **(B)**, the tessellation overlay map superimposed on the original image to demonstrate the spatial distribution of detected tessellated areas **(C)**, and the binary segmentation map derived from the trained model that was subsequently used for quantitative calculation of FTD **(D)**.

In 2021, Shao et al. introduced a novel AI-based image processing technique to quantitatively extract the average choroidal exposure area per unit area, referred to as fundus tessellated density (FTD), from color fundus photographs (57). The study included 3,468 residents aged 50 and above from five communities in Beijing, with comprehensive acquisition of ophthalmic clinical parameters. Through image preprocessing, manual annotation, and segmentation via deep learning models, the FTD was quantitatively extracted in an automated manner (Figure 1). Multivariable linear regression demonstrated that FTD was significantly associated with several demographic and ocular parameters, including age, sex, body mass index (BMI), axial length, subfoveal choroidal thickness, and peripapillary atrophy. For instance, FTD exhibited an approximate 33.1% increase per decade of age. Notably, myopic individuals exhibited markedly higher FTD values than emmetropic and hypermetropic individuals, with FTD correlating strongly with lower spherical equivalent (SE) values, longer axial lengths, and larger areas of peripapillary atrophy. Distinct FTD distribution patterns were observed across varying refractive severities, with mild (−0.50 to > −3.00 D), moderate (−3.00 to > −6.00 D), and high (≤ −6.00 D) myopia exhibiting progressively greater spatial involvement (58) (Figure 2). As FTD reflects the degree of choroidal exposure, it has been proposed as a potential quantitative biomarker for choroidal thickness in population-based studies, contributing to risk stratification for myopia-related structural alterations.

With the growing application of FTD quantification in myopia research, subsequent studies have further explored image-based retinal analysis using data-driven approaches. In 2023, Li et al. evaluated FTD in a cohort of 1,084 university students, reporting significant associations between higher FTD values and increased choriocapillaris vessel density, along with decreased deeper choroidal vessel density (59). These associations may reflect underlying alterations in choroidal perfusion and vascular permeability. Regional heterogeneity in FTD distribution was noted, with significantly higher values in the nasal quadrant relative to the superior, inferior, and temporal regions, underscoring topographic differences across the posterior pole. Subsequently, FTD quantification has been applied to children and adolescents. Huang et al. (32)conducted a study on 1,062 children born between September 2011 and August 2012, finding that the prevalence of FTs in the entire retina, macula, and peripapillary regions ranged from 42.18% to 49.72%. ROC curve analysis indicated that the critical value of FTD exhibited high sensitivity, specificity, and Youden’s index, all of which increased significantly with the severity of FT. In a contemporaneous study, Huang et al. demonstrated that when FTD concentrated around the optic disk, this distribution pattern was more strongly associated with myopia-related fundus changes, such as peripapillary atrophy and choroidal thinning (60). In a 2025 follow-up study, Huang further expanded this line of research by applying deep learning techniques to quantify FTD in children aged 6–15 years, exploring the relationship between the distribution of FTs in the macula and peripapillary regions and refractive status. The findings revealed that macular FTD distribution was closely associated with longer axial length and more severe myopic refractive error, and this pattern was more frequently observed among myopic individuals. These results suggest that macular FTD distribution may serve as a valuable imaging biomarker for identifying individuals at elevated risk of myopia progression (61) (Table 1).

**TABLE 1:** Summary of studies applying artificial intelligence–assisted quantification of fundus tessellation related to myopia.

References	*N*	Region	Age	Image type and coverage	Objective	Key findings/significance
(57)	3,468	Beijing, China	>50 (64.1 ± 9.7)	45° macula-centered fundus photo	Quantify FTD and associated factors using AI	First AI-based quantitative FTD analysis in older adults
(32)	1,062	Nanjing, China	7.38 ± 0.29 (6.83–7.83)	45° fundus; macular + 6 mm + 4 mm peripapillary	AI screening of FT in children	Defined FTD thresholds; early detection of severe FT in children
(76)	14,234	Shanghai, China	12.01 ± 4.17 (4–18)	ETDRS 6 mm macular grid (9 zones)	FTD distribution and myopic maculopathy correlation	FTD reflects severity; nasal zone most sensitive; FTD ≥ 2.22% critical
(59)	1,002	Qingdao, China	19.32 ± 1.11 (17–23)	6 mm macular grid divided into Sup, Inf, Nas, Tem	FTD assessment in young adults	AI-derived FTD aids in objective retinal evaluation
(62)	407	Beijing, China	52 ± 7 (13–83)	45° macula-centered fundus	Evaluate FTD across pathological myopia subtypes	FTD declines with PM severity; may refine PM classification
(77)	1776	Mojiang, China	7.71 ± 0.55	Handheld 45° fundus camera	Link FTD with AL/SE progression (4 years)	FTD predicts axial elongation and SE progression
(78)	1,907	Haikou, China	10.6 ± 2.33 (7–14)	45° color fundus	AI recognition of FT in school screening	High AI sensitivity/specificity; useful for screening
(79)	584 (dev), 599 (val)	Qingdao, China	24.44 ± 5.59	45° macula-centered fundus	FTD and optic disc morphology correlation	FT/OD highly correlate with myopia severity
(74)	3,468	Beijing, China	>50 (64.1 ± 9.7)	45° macula-centered fundus	ML prediction of FT severity	FTD thresholds predict FT grades for screening
(61)	1942	Beijing, China	Same as above	45° fundus photographs	Compare FT patterns: macular vs. peripapillary	Macular pattern more myopia-related; AI-enhanced classification
(32)	577	Nanjing, China	7.44 ± 0.28	45° macula-centered fundus	Analyze FT pattern and subfoveal choroid	Peripapillary FT pattern relates to myopic changes
(80)	206	Guangzhou, China	HM: 47 ± 12; HMG: 42 ± 13	45° macula-centered fundus	Evaluate FTD in high myopia vs. glaucoma	FTD as biomarker for glaucomatous changes in HM

Furthermore, artificial intelligence–assisted quantitative approaches have introduced novel perspectives into the assessment of fundus tessellation, particularly in the objective grading of its severity. Compared to traditional manual FT grading methods (22), AI offers more accurate and objective evaluation tools. For instance, He et al. utilized artificial intelligence to quantitatively assess FTD from fundus photographs across various stages of pathological myopia, focusing on both macular and peripapillary regions (62). Their findings revealed a decreasing trend in FTD values across different stages of pathological myopia. A significant innovation of this study was the automated identification and quantification of patchy atrophic areas in the fundus, which allowed for precise comparison with remaining FTD regions to better understand the extent and characteristics of atrophic changes in pathological myopia.

Overall, the integration of artificial intelligence into quantitative fundus tessellation analysis not only enhances the precision and objectivity of FT evaluation but also opens promising avenues for advancing research on myopia and its related retinal alterations.

## Discussion

This review provides an integrative overview of recent advances in FT quantification in myopia research. Pigmentary changes in the fundus, resulting from increased visibility of choroidal vasculature near the macula and arcuate vessels, represent early manifestations of retinal–choroidal structural remodeling during myopia development (18). Accumulating evidence indicates that FT is highly prevalent across different populations and age groups and demonstrates significant associations with key myopic parameters, including refractive status, axial length, visual acuity, and choroidal thickness (19). Longitudinal observations further suggest that FT may precede more advanced myopic maculopathy, highlighting its potential value in early screening, risk stratification, and disease monitoring (7). As a result, FT is gaining attention for its potential role in clinical screening, risk stratification, and early intervention. Although experienced ophthalmologists can generally detect early FT, challenges persist in retinal image analysis and grading, particularly regarding issues of consistency and sensitivity. These diagnostic limitations are further compounded by technical variables including ocular positioning, illumination conditions, and imaging device specifications, all of which may affect image quality and complicate FT assessment (37). Given the potential predictive value of FT in myopia progression, there is a pressing need for more objective and standardized quantitative methods to enhance its clinical applicability and research value.

Recent advancements in AI have significantly transformed diagnostic approaches in ophthalmology, particularly in medical image analysis. Deep learning-based models have been widely validated and are progressively being applied in clinical settings. For instance, the Retinal Artificial Intelligence Diagnostic System (RAIDS), developed by Dong et al., can automatically identify ten common retinal diseases, including diabetic retinopathy and macular degeneration. In a prospective large-scale dataset (208,758 fundus images from 110,784 individuals), the system demonstrated an average sensitivity of 89.8%, with diagnostic accuracy ranging from 95.3% to 99.9%. Its performance was comparable to that of experienced retinal specialists, while reducing image evaluation time by over 95% (63). In the context of myopia, AI has also shown substantial promise, advancing diagnostic capabilities and research. For children, AI applications focus on predicting myopia progression and optimizing interventions, such as predicting the risk of high myopia (64, 65) and adjusting orthokeratology treatments based on corneal parameters or topography (66, 67). In adults, AI is primarily used to detect and classify pathologic myopia and myopia-related complications (68, 69). Notably, conventional FT grading relies heavily on subjective visual interpretation, making it susceptible to inter-observer and intra-observer variability and limiting reproducibility across imaging conditions. In contrast, AI-based quantitative pipelines extract image features in a standardized and automated manner, substantially improving measurement consistency while reducing human-related variability (70). Additionally, the high-throughput capacity of deep learning models enables rapid and scalable analysis of large imaging datasets, which is essential for population-based myopia screening (71). Importantly, AI algorithms are capable of identifying subtle textural or chromatic changes—such as early increases in choroidal visibility—that may precede clinically evident alterations, thus offering the potential for earlier risk stratification (72). These advantages collectively address key limitations of traditional FT evaluation and underscore the value of AI-driven approaches for more objective and sensitive assessment. While AI has demonstrated strong diagnostic performance across various retinal conditions, its application in myopia has predominantly focused on qualitative lesion recognition rather than quantitative characterization of early structural changes. Consequently, the development of robust AI algorithms capable of delivering objective, consistent, and high-throughput FT measurements remains a critical and timely research priority.

Building on the advancements in AI-driven FT quantification, its application in other imaging modalities, such as optical coherence tomography (OCT) and color fundus photographs, has also shown promising results in ophthalmology. While OCT is limited to measuring choroidal thickness within specific macular regions or scanning areas, color fundus photographs provide broader retinal coverage, offering more comprehensive structural information with higher research value and clinical potential. Quantitative analysis of FT using color fundus images not only addresses the subjectivity inherent in traditional qualitative assessments, but also offers a comprehensive framework for evaluating the spatial distribution, severity, and progression-related correlations of FT in myopia. Since Shao et al. first introduced FTD in 2021—quantifying the proportion of choroidal exposure per unit area from color fundus photographs—this method has become a key tool for objective FT evaluation (59). Deep learning segmentation models are used to accurately identify and separate retinal vessels, the optic disk, and other interfering structures, allowing the quantification of choroidal exposure in the background area and providing a numerical indicator of FT severity. FTD-based studies have been conducted across diverse populations—including elderly individuals, university students, and children—demonstrating its applicability and relevance in different age groups and stages of myopia development. For example, research on FTD in seven-year-old children found that FT is a common retinal change (32), with significant associations between FTD and myopia-related parameters, such as axial length, choroidal thickness, and refractive status. These findings may be linked to pathological myopia changes, such as the correlation between FTD and increased spherical equivalent, which may be explained by choroidal atrophy in myopic eyes, particularly in high myopia (73). Furthermore, machine learning models that incorporate FTD and other factors to predict the severity of fundus tessellation have demonstrated good classification accuracy (74). Alternatively, direct quantitative assessment of FTD across different types of pathological myopia may provide insights for refining current PM classification systems (62).

As research continues to expand the clinical applicability of FTD, some intriguing observations have emerged. One is the clinical significance of the spatial distribution of FTD and its relative position. Previous studies have found that choroidal thickness is unevenly distributed across the retina, with the nasal region being significantly thinner than the temporal region (20, 31). Consistent with this, studies analyzing FTD have observed significantly higher FTD values in the nasal region compared to the superior, inferior, and temporal regions. As myopia worsens and axial elongation increases, the nasal region is more prone to stretching and atrophic structural changes. This observation not only aligns with anatomical characteristics of the choroid (29) but also suggests that the spatial distribution and relative position of FTD could serve as important indicators of the severity of myopic retinal changes. Additionally, differences in segmentation methods have been observed. The “X” segmentation method shows significant correlations between FTD in each region (superior, inferior, nasal, and temporal) and total FTD, whereas the “+” method does not show significant correlations for all quadrants. This discrepancy arises from the varying spatial orientations of the segmentation methods and their clinical applications. For instance, the “X” method is more suitable for evaluating the morphology of the optic disk border, while the “+” method is better suited for detecting retinal nerve fiber layer defects in glaucoma patients (75). Taken together, quantitative analysis based on FT offers a novel method for assessment and also provides new insights into its clinical significance with respect to spatial distribution and segmentation strategies.

While substantial progress has been made in FT quantification, several critical limitations continue to hinder its broader application. First, most existing studies are based on single-center hospital populations with limited sample sizes, and few have undergone multicenter or cross-population validation, which still constrains the generalizability and robustness of current FT quantification models. Second, many studies are cross-sectional, lacking longitudinal follow-up data to identify dynamic patterns in FT during myopia onset and progression. As such, the temporal relationship between FTD and pathological changes at different stages of myopia remains unclear, hindering its potential for risk prediction and disease monitoring. Third, although deep learning–based segmentation algorithms generally perform well, heterogeneity in training datasets, image quality control, and segmentation strategies can introduce variability in FTD estimation. Variations across imaging devices and acquisition conditions, together with ethnic differences in fundus pigmentation, may further influence model behavior and limit cross-population generalizability. Furthermore, current models exhibit limited cross-domain generalizability, and their performance often decreases when applied to datasets from different imaging devices, clinical centers, or ethnic populations; future work incorporating transfer learning and domain-adaptation strategies may help improve robustness across diverse settings. Moreover, dataset imbalance—particularly the under-representation of early or mild FT cases—can lead to biased model training, thereby reducing robustness when deployed in broader clinical environments. Besides, current FT quantification lacks standardization across studies. Different metrics—such as FTD, TFI, and MCDI—use heterogeneous region definitions and color–intensity normalization strategies, making cross-study comparisons difficult and highlighting the need for unified FT measurement criteria. Additionally, the lack of interpretability in algorithmic decision-making limits its clinical reliability and acceptance.

In conclusion, with the rapid development of AI technology, FTD has emerged as a reliable and objective quantitative index for fundus tessellation, demonstrating strong practical value in both epidemiological studies and clinical evaluations. Its spatial distribution characteristics, associations with key ocular parameters, and regional analytical significance provide new insights into myopia-related structural alterations. However, despite these advances, several challenges remain, including the lack of standardized FT metrics, limited multicenter validation, and the need for improved generalizability across imaging devices and populations. Looking forward, multimodal AI approaches that integrate fundus photography, OCT, and clinical data may enhance the prediction of myopia progression, while incorporation of automated FT analysis into teleophthalmology platforms could support large-scale population screening. Ethical and regulatory considerations—particularly algorithmic transparency and mitigation of data bias—will also be essential for the clinical adoption of AI-based FT assessment. Overall, these advances highlight the substantial potential of AI-driven FT quantification in elucidating the structural mechanisms of myopia and improving early identification of individuals at risk.
